# Difference in the efficacy of intravitreal dexamethasone implant before and after silicone oil removal: A case report

**DOI:** 10.1097/MD.0000000000025161

**Published:** 2021-03-19

**Authors:** Jae Hong An, Yu Cheol Kim

**Affiliations:** Department of Ophthalmology, Keimyung University Dongsan Medical Center, Daegu, Republic of Korea.

**Keywords:** dexamethasone, efficacy, intravitreal implant, macular edema, silicone oil

## Abstract

**Rationale:**

An intravitreal dexamethasone (IV-DEX) implant is safe and effective for the treatment of macular edemas; however, the efficacy of IV-DEX implants in silicone oil (SO)-filled eyes remains controversial. There is no previous study comparing an IV-DEX implant in the same eye with and without intravitreal SO.

**Patient concerns:**

A 72-year-old man with proliferative diabetic retinopathy, macular edema, and rhegmatogenous retinal detachment, treated with pars plana vitrectomy with SO tamponade had refractory macular edema.

**Diagnosis:**

Refractory macular edema.

**Intervention:**

Subtenon triamcinolone injection, intravitreal anti-vascular endothelial growth factor injection, and IV-DEX implantation were performed; this was followed by intravitreal SO removal combined with IV-DEX implantation.

**Outcomes:**

The macular edema did not decrease significantly with posterior subtenon triamcinolone injection, intravitreal anti-vascular endothelial growth factor injection, and IV-DEX implantation; however, the edema was relieved after SO removal and a new IV-DEX implantation.

**Lessons:**

IV-DEX implant may be less efficacious in the treatment of macular edema in an SO-filled eye than that in a normal vitreous cavity.

## 1 Introduction

An intravitreal dexamethasone implant (IV-DEX implant; Ozurdex, Allergan, Inc., CA, USA) for the sustained release of dexamethasone (700 μg) is safe and effective for the treatment of macular edemas in patients with various retinal conditions.^[1,2]^ Eyes with silicone oil (SO) often have refractory macular edema, which is considered curable with a IV-DEX implant; however, the efficacy of IV-DEX implants in SO-filled eyes remains controversial.^[3–5]^ To our best knowledge, no study has compared the efficacy of an IV-DEX implant in the same eye with and without SO. Here, we report the variable efficacy of an IV-DEX implant before and after SO removal.

## 2 Case report

**Figure 1:**
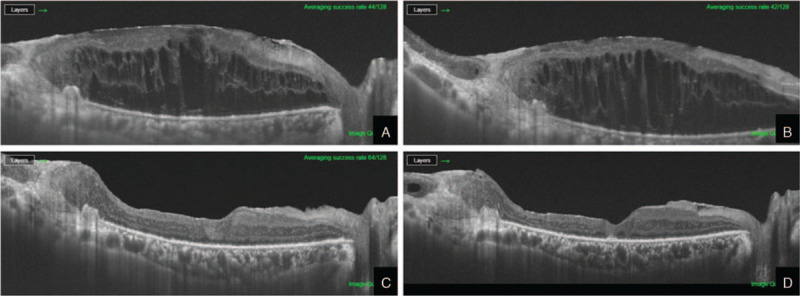
Optical coherence tomography images of the horizontal line scans through the macula of the patient's right eye. (A) Before the implantation of the intravitreal dexamethasone (IV-DEX) implant (central subfield macular thickness [CSMT], 860 μm). (B) One month after the implantation of the IV-DEX implant in the silicone oil-filled vitreous cavity (CSMT, 786 μm) (C) One month after the implantation of the IV-DEX implant after silicone oil removal (CSMT, 301 μm). (D) Fourteen months after implantation of the second IV-DEX implant, which was 2 months after the third implantation (CSMT, 229 μm).

A 72-year-old man with a history of diabetes and hypertension visited our hospital with a chief complaint of right vision deterioration. His left eye was diagnosed with postoperative endophthalmitis by pars plana vitrectomy (PPV) at a local ophthalmic clinic 1 month prior; therefore, he underwent an immediate PPV with SO tamponade at our hospital. Postoperatively, his best-corrected visual acuity (BCVA) ended with non-light perception. His right eye was pseudophakic with a BCVA of 20/400 (Snellen chart) and intraocular pressure of 13 mm Hg (applanation tonometry), and a history of PPV for proliferative diabetic retinopathy and diabetic macular edema. The macula of his right eye was edematous and detached with a 1/5 disc-diameter-sized retinal break, which was 3-disc-diameter temporal to the fovea on optical coherence tomography imaging and fundus examination. In addition, the vitreous cavity was filled with SO. After another PPV with SO tamponade, the retina was completely reattached; however, the macular edema persisted postoperatively. The macular edema did not decrease despite posterior subtenon triamcinolone injection and intravitreal anti-vascular endothelial growth factor injection (Fig. 1a); therefore, he was treated with an IV-DEX implant. One week later, the implant was identified at the far peripheral fundus. The central subfield macular thickness (CSMT) had decreased slightly from 860 μm to 786 μm over a month (Fig. 1b). Three months after the first IV-DEX implant, the SO was removed and another IV-DEX implant was placed at the end of the surgery. One month after the SO removal, the macular edema had relieved, and the CSMT was 301 μm. This decrease remained stable postoperatively even at 14-month follow-up without additional treatment except an additional IV-DEX implant 1-year postoperatively (CSMT at postoperative 14 months, 229 μm). Moreover, the patient's BCVA improved to 40/200 (Fig. 1c and d). There were no postoperative complications during the follow-up period.

## 3 Discussion

This case report shows that macular edema refractory to posterior subtenon triamcinolone injection, intravitreal anti-vascular endothelial growth factor administration, and an IV-DEX implant in an eye with SO was effectively relieved with the IV-DEX implant only after SO removal.

Previous case studies have reported contrasting findings on the behavior and efficacy of IV-DEX implants in eyes with a SO-filled vitreous cavity. Flores Villalobos et al reported that in vitro, DEX implants had less anti-inflammatory effects and more irregularity in its levels in SO than those in saline solution, and suggested that relatively denser mediums alter the pharmacokinetics of the IV-DEX implant; therefore, the IV-DEX implant should not be used in dense SO-filled eyes.^[3]^ However, Afshar et al and Esenulku et al reported that the IV-DEX implant trapped at the macula in an SO-filled eye could improve BCVA and relieve macular edema.^[4,5]^ In light of these previous reports, the pharmacological effects of an IV-DEX implant is presumed to be more sensitive to its proximity to the target tissues. In contrast, Kim et al reported that an IV-DEX implant in an SO-filled eye with macular edema secondary to chronic non-infectious uveitis was efficacious in improving BCVA and CSMT, regardless of the implant's proximity to the macula; the efficacy in the previous study might have decreased because of inflammation and not by the direct effect of DEX on the macula.^[6]^ An IV-DEX implant rarely localizes to the macula because of the buoyancy of SO, and moreover, its location hardly changes in SO-filled eyes until it disappears.

In this case, the first IV-DEX implant was not efficacious presumably because it was not sufficiently close to the macula to deliver a therapeutic level of DEX through the SO. However, after SO removal, the second IV-DEX implant may have stably delivered a therapeutic dose of DEX to the macula without any hindrance.

In conclusion, this case shows that the efficacy of IV-DEX implant differs before and after SO removal. Moreover, the IV-DEX implant is less efficacious in the treatment of a macular edema in a SO-filled eye than that in a normal vitreous cavity, and especially when the implant is not proximal to the macula.

## Author contributions

**Conceptualization:** Yu Cheol Kim.

**Data curation:** Jae Hong An, Yu Cheol Kim.

**Investigation:** Jae Hong An.

**Methodology:** Jae Hong An, Yu Cheol Kim.

**Supervision:** Yu Cheol Kim.

**Visualization:** Yu Cheol Kim, Jae Hong An.

**Writing – original draft:** Jae Hong An.

**Writing – review & editing:** Yu Cheol Kim.
